# Dysregulation of Wnt signaling in bone of type 2 diabetes mellitus and diabetic Charcot arthropathy

**DOI:** 10.1186/s12891-022-05314-9

**Published:** 2022-04-18

**Authors:** Laurens Christian Gassel, Sandra Schneider, Ingo Jörg Banke, Karl Friedrich Braun, Christoph Volkering, Leonie Zeeb, Rainer Hans Hermann Burgkart, Rüdiger von Eisenhart-Rothe, Peter Biberthaler, Martijn van Griensven, Alexander Tobias Haug

**Affiliations:** 1 Department of Experimental Trauma Surgery, and Department of Surgery, Klinikum rechts der Isar, Technical University of Munich, Ismaninger Strasse 22, 81675 Munich, Germany; 2 Department of Orthopedics and Sports Orthopedics, Klinikum rechts der Isar, Technical University of Munich, Ismaninger Strasse 22, 81675 Munich, Germany; 3 Charité – Berlin University of Medicine, Center for Musculoskeletal Surgery, Campus Virchow-Klinikum (CVK), Augustenburger Platz 1, 13353 Berlin, Germany; 4 Department of Trauma Surgery, Klinikum rechts der Isar, Technical University of Munich, Ismaninger Strasse 22, 81675 Munich, Germany; 5 Orthoevo, Martiusstrasse 3, 80802 Munich, Germany; 6 Department cBITE, MERLN Institute, Maastricht University, Universiteitssingel 40, 6229 ER Maastricht, the Netherlands; 7 Department of Experimental Trauma Surgery and, Department of Orthopedics and Sports Orthopedics, Klinikum rechts der Isar, Technical University of Munich, Ismaninger Strasse 22, 81675 Munich, Germany

**Keywords:** Diabetic bone disease, Type 2 diabetes mellitus, Wnt signaling, TCF7L2, Osteocalcin

## Abstract

**Background:**

Type 2 diabetes mellitus (T2DM) patients show a markedly higher fracture risk and impaired fracture healing when compared to non-diabetic patients. However in contrast to type 1 diabetes mellitus, bone mineral density in T2DM is known to be normal or even regionally elevated, also known as diabetic bone disease. Charcot arthropathy is a severe and challenging complication leading to bone destruction and mutilating bone deformities. Wnt signaling is involved in increasing bone mineral density, bone homeostasis and apoptotic processes. It has been shown that type 2 diabetes mellitus is strongly associated with gene variants of the Wnt signaling pathway, specifically polymorphisms of TCF7L2 (transcription factor 7 like 2), which is an effector transcription factor of this pathway.

**Methods:**

Bone samples of 19 T2DM patients and 7 T2DM patients with additional Charcot arthropathy were compared to 19 non-diabetic controls. qPCR analysis for selected members of the Wnt-signaling pathway (WNT3A, WNT5A, catenin beta, TCF7L2) and bone gamma-carboxyglutamate (BGLAP, Osteocalcin) was performed and analyzed using the 2-ΔΔCt- Method. Statistical analysis comprised one-way analysis of variance (ANOVA).

**Results:**

In T2DM patients who had developed Charcot arthropathy WNT3A and WNT5A gene expression was down-regulated by 89 and 58% compared to healthy controls (*p* < 0.0001). TCF7L2 gene expression showed a significant reduction by 63% (*p* < 0.0001) and 18% (*p* = 0.0136) in diabetic Charcot arthropathy. In all diabetic patients BGLAP (Osteocalcin) was significantly decreased by at least 59% (*p* = 0.0019).

**Conclusions:**

For the first time with this study downregulation of members of the Wnt-signaling pathway has been shown in the bone of diabetic patients with and without Charcot arthropathy. This may serve as future therapeutic target for this severe disease.

**Supplementary Information:**

The online version contains supplementary material available at 10.1186/s12891-022-05314-9.

## Background

### Type 2 diabetes mellitus and bone

Diabetes mellitus is a growing global health burden. The worldwide prevalence of diabetes mellitus is estimated at around 8.8% [1]. In contrast to type 1 diabetes mellitus, the majority of publications concerning diabetic bone disease describe increased bone mineral density (BMD) in type 2 diabetes mellitus (T2DM), even after adjusting for body mass index (BMI) and age [2, 3]. At the same time, T2DM is associated with an increased fracture risk [4–6]. Furthermore, T2DM is accompanied by an impaired bone healing after fracture [7]. A plausible structural anatomic explanation of the paradox of higher BMD and increased risk of fracture may be alterations in bone microarchitecture [8, 9].

### Charcot arthropathy: a severe diabetic complication in bone

Within the course of T2DM, complications such as angiopathy, nephropathy and polyneuropathy develop. On the basis of diabetic polyneuropathy, the feared Charcot arthropathy may develop, named after the French neurologist Jean-Martin Charcot [10–12]. Charcot arthropathy is characterized by a stepwise difficult to treat development from fractures, dislocations, and bone erosions over hypertrophic repair to finally total deformation and functional insufficiency. Heavy reconstructive operations or even amputation of the affected limb may be required [10, 11]. Despite the severe impact on the affected patients, the pathophysiology of diabetic bone disease and Charcot arthropathy still is not fully understood [12, 13].

### Gene variants of the Wnt pathway are associated with diabetes mellitus

In T2DM, gene variants of the members involved in Wnt signaling have been shown to be strongly associated with the development of the disease [14, 15]. Especially polymorphisms of transcription factor 7 like 2 (TCF7L2) seem to contribute to T2DM. A mechanism, by which risk alleles of TCF7L2 increase the risk of T2DM and include an impaired insulin secretion in response to oral glucose uptake in the pancreatic gland [16, 17]. Interestingly, there is some evidence that the bipartite transcription factor complex catenin beta/TCF may act as an effector for other signaling pathways such as insulin, insulin-like growth factor-1 and glucagon-like peptide-1 [18].

**Fig. 1:**
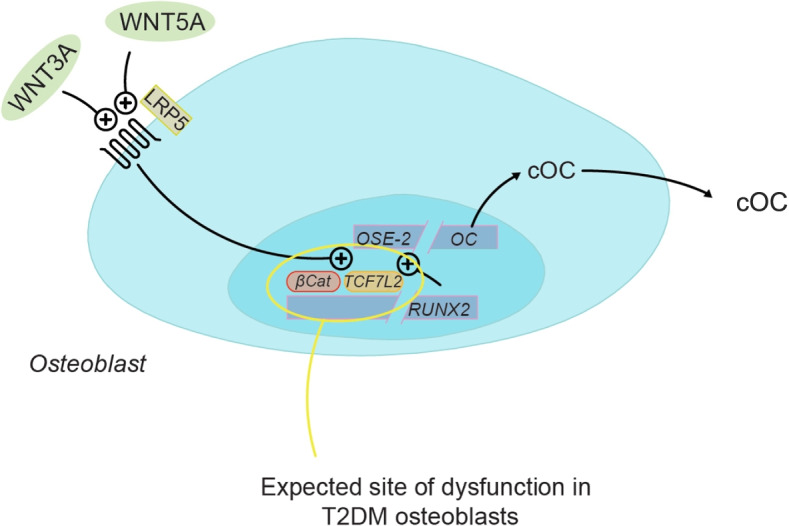
Wnt signaling pathway regulating the expression of osteocalcin in osteoblasts

Wnt ligands are secreted glycoproteins, which exert their function via intracellular signaling pathways (simplified depiction see Fig. 1). Within the framework of the canonical Wnt pathway, these glycoproteins bind to seven-transmembrane domain Frizzled receptors and their co-receptors - low-density lipoprotein receptor-related protein 5/6 (LRP5/6). In this liganded state, Dishevelled associates with the Wnt receptor, which causes the disruption of a destruction complex consisting of adenomatous polyposis coli, axin and glycogen synthase kinase 3 beta /casein kinase 1 alpha. In this case, catenin beta remains hypophosphorylated (active), translocates into the nucleus and recruits a co-factor of the TCF family, for instance TCF7L2. The bipartite transcription factor complex subsequently regulates gene expression of various target genes. In the unliganded state of Wnt receptors, the destruction complex phosphorylates catenin beta, followed by ubiquitination and eventually proteasomal degradation [15]. Interestingly, insulin signaling may enhance Wnt signaling via IRS (insulin receptor substrate) and mediate stabilization of Dishevelled [19].

### Wnt signaling in bone

In bone, the Wnt signaling pathway is responsible for the increase of bone density. Amongst others, this is achieved by induction of differentiation and inhibition of apoptosis of osteoblasts [20–24]. Osteoblasts synthesize and organize bone matrix. Mutations of LRP5, the co-receptor of Wnt signaling, are associated with changes in bone biology, e.g.in Hyperostosis corticalis generalisata congenita. The latter patients develop abnormal high bone density [15]. Furthermore, these patients showed distinctly elevated levels of serum BGLAP (following denominated as osteocalcin), a marker of bone formation [25].

Osteocalcin - a protein primarily expressed in osteoblasts – on the other hand seems to modulate pancreatic beta cell function [26]. Insulin signals in osteoblasts activate osteocalcin that promotes glucose metabolism [27].

We hypothesize a causal connection between Wnt signaling pathway and diabetic bone disease and Charcot arthropathy. Therefore, we investigated for the first time to our knowledge gene expression of the Wnt signaling pathway in bone from patients suffering from type 2 diabetes mellitus and diabetic Charcot arthropathy. We focused on studying a limited number of Wnt signaling members that play key roles in osteoblast biology, differentiation and are especially well studied in bone cells in vitro. Wnt3a represents a classical ligand of the canonical pathway and Wnt5a is a ligand of non-canonical pathways but may also act through the canonical pathway [28–30].

## Methods

### Patient selection

The study was conducted in accordance with the Declaration of Helsinki. A positive vote of the ethics committee of the Technical University of Munich was obtained (No. 5579/12), as well as written informed patient consent. All experimental protocols were approved by the ethics committee of the Technical University of Munich.

19 T2DM patients were included. Inclusion criteria was the full extent of metabolic syndrome with abdominal obesity (waist circumference ≥ 94 cm [male] or ≥ 80 cm [female]), hypertriglyceridemia (> 150 mg/ dl), HDL cholesterol level of < 40 mg/ dl [male] or < 50 mg/ dl [female], hypertension (RR ≥ 140/90 mmHg), and already diagnosed T2DM according to WHO criteria [31]. Samples were collected during operation of fractures of the lower limb.

Seven additional patients with T2DM and Charcot arthropathy were included undergoing external fixation surgery of the Charcot foot. All Charcot arthropathy patients suffered from great pain, loss of function of the foot and radiological evidence of skeletal destruction according to Eichenholtz classification [10, 32].

Nineteen patients were included in the non-diabetic control group within the context of fracture surgery of the lower limb after showing no clinical and laboratory signs of diabetes.

Exclusion criteria were causes of secondary osteoporosis (i.e. neoplasia, hepatic insufficiency, renal insufficiency, organ transplantation), local and systemic infections and the use of glucocorticoids, antiepileptic drugs, immunosuppressants.

### Bone samples

The intraoperatively resected bone samples were transferred into 15 ml tubes within 60 min after extraction. The tubes were filled with 2–3 ml “Allprotect Tissue Reagent” (Qiagen N.V., Hilden, Germany) completely covering the fragments, snap frozen and stored at − 80 °C until further processing.

### Isolation of RNA

For RNA extraction the phenol-chloroform method followed by ethanol precipitation was used. First, a 5 mm cancellous bone fragment was mechanically ground using a bench-top ball mill (MM 400, Retsch GmbH, Haan, Germany). The container of the mill with the cancellous fragment was cooled for 2 min in liquid nitrogen to allow the finest possible comminution of the fragment and to avoid RNA degradation. The grinding itself was carried out for 30 s at a frequency of 30 Hz. 1 ml of TRI reagent (Sigma-Aldrich, St. Louis, Missouri, USA) was added, and the solution then transferred to a 2 ml reaction tube. After incubation of the suspension on ice for 5 min, 200 μl of chloroform was added. After mixing on the vortex mixer, the suspension was incubated on ice for 10 min, followed by centrifugation (14,000 g, 10 min, 4 °C). The aqueous upper phase (containing total RNA) was transferred into a 2 ml reaction tube filled with 500 μl of isopropanol. The sample suspension was incubated on ice for 10 min and centrifugation was performed at 14,000 g for 10 min at 4 °C. Total RNA was extracted, remained as a pellet at the bottom of the reaction tube and the supernatant was discarded. 1 ml of 70% ethanol was used for washing and centrifuging (14,000 g, 10 min, 4 °C.) of the RNA pellet. This step was repeated once before the pellet was finally resuspended in 30 μl water and stored at − 80 °C.

### cDNA synthesis

For processing of isolated RNA strands complementary DNA (cDNA) strands were produced. Eppendorf MasterCycler S nexus (Eppendorf AG, Hamburg, Germany) and the “First Strand cDNA Synthesis Kit” (Thermo Fisher Scientific Inc., Waltham, Massachusetts, USA) following the manufacturer’s instructions were used. According to the previously measured RNA content, a maximum of 5 μg RNA - as determined by photometric measurement (BioPhotometer 6131, Eppendorf AG, Hamburg, Germany) - of a sample was mixed with 1 μl oligo (dT)_18_ Primer and 1 μl Random Hexamer Primer in a PCR tube. The tube was ice-cooled on a rack throughout the process. The tube was filled up to a volume of 11 μl with water, heated up in the MasterCycler S for 5 min at 65 °C and subsequently cooled down to 4 °C. This was followed by adding 4 μl 5X Reaction Buffer, 1 μl RiboLock® RNase Inhibitor (20 U/μl), 2 μl dNTP Mix (10 mM) and 2 μl M-MuLV Reverse Transcriptase (20 U/μl). The synthesis of cDNA occurred at 37 °C for 60 min. The reaction was then terminated by heating the tubes to 70 °C for 5 min. After cooling the synthesized cDNA to 4 °C, the samples were diluted to a concentration of 10 ng/μl by the addition of water.

### qPCR

Quantitative PCR (q-PCR) was performed using the SsoFast EvaGreen Supermix and the real-time-PCR-Cycler CFX 96 touch (both BioRad Laboratories Inc., Hercules, USA) following the manufacturer’s instructions. q-PCR was performed for WNT3A, WNT5A, catenin beta, TCF7L2, osteocalcin, type 1 collagen (COL1A1) and fibronectin. A master mix was prepared, containing the following reagents: 10 μl 2xSsoFast™ EvaGreen® Supermix, 0.8 μl forward primer (5 μM), 0.8 μl reverse primer (5 μM). The calculated amount of required cDNA and the reaction suspension was filled up with RNAse-free water to a total volume of 20 μl. The following reaction conditions had been established: Enzyme activation by heating to 98 °C for 3 min, followed by 40 cycles of denaturation (98 °C for 10 s) and subsequently annealing at primer-specific temperature for 15 s (see Table 1 in supplementary material). The melting curve was detected by heating from 55 °C to 95 °C. The primers were obtained from Eurofins Genomics Germany GmbH, Ebersberg, Germany.

The PCR data were analyzed using the 2^-ΔΔ*CT*^ method. In order to standardize the expression levels, the threshold cycle (C_T_) of gene, sequences was normalized against the corresponding C_T_ of actin beta, a housekeeper gene. The obtained ΔC_T_ values were normalized to a matched, healthy control – according to lowest difference in gender and age. Therefore, in the depiction of the results, the healthy controls are equal 1.0 in order to demonstrate the relative expressions of the target genes in relation to the control group. Thus, the relative expression of target genes detected by qPCR are presented [33].

### Statistical analysis

In order to determine the required sample size for the experiments, we used the previously obtained data from our in vivo study analyzing different gene expression in bone of patients suffering from T2DM [34]. As an effect size of the past experiments, we obtained a range of values from 0.44 to 1.89. Therefore, we decided to define the effect size d as 0.9; α = 0.05, Power 0.8. Before further statistical analyses, outliers were identified objectively using Robust regression and Outlier removal (ROUT; adapted algorithm for column data); Q = 2%, which corresponds to a rather conservative outlier removal. One-way analysis of variance (ANOVA) on ranks Kruskal-Wallis test and Dunn’s multiple comparisons test were performed. The confidence interval was 95%. The statistically significant *p*-value was set as *p* ≤ 0.05 and is reported as exact *p* value for the single results. The software program used for statistical analyses and creating the graphs is GraphPad Prism Software 8.1.2, El Camino Real, USA. The results are depicted as boxplot with marked median, interquartile range (between 25 and 75%) and whiskers of the max./min. Values.

## Results

### Patients’ characteristics

The T2DM group showed a median age of 78.2 years, the non-diabetic comparison group a median age of 73.4 years. The subgroup of diabetic patients with Charcot arthropathy was younger with a median age of 59.1 years. The median BMI of the healthy controls was 25.9 kg/m^2^,the T2DM group showed a BMI of 31.4 kg/m^2^ and the Charcot arthropathy group a BMI of 32.2 kg/m^2^. Therefore, the healthy controls were slightly overweight and the T2DM as well as the Charcot patients obese. Median HbA1c was 5.3% in the control, 6.6% in T2DM and 6.2% in the Charcot arthropathy group (the normal range is 4.8–5.9%), the median fasting glucose concentration 88.0 mg/dl as compared to 164.0 mg/dl and 132.2 mg/dl (the normal range is 70–110 mg/dl), respectively (see Table 1).

**Table 1:** Patients’ characteristics of the control group, T2DM group and Charcot arthropathy group

Characteristic [unit; range]	Control Group	T2DM Group	Charcot Arthropathy Group
Sex	13 ♀ (68%)	9 ♀ (47%)	3 ♀ (43%)
	6 ♂ (32%)	10 ♂ (53%)	4 ♂ (57%)
Age [years]	73.4	78.2	59.1
BMI [kg/m^2^]	25.9	31.4	32.2
Fasting glucose [70–110 mg/dl]	89.8	164.0	132.2
HbA1c [< 5.7%]	5.3	6.9	6.2
Triglycerides [≤200 mg/dl]	112.9	204.8	135.5
HDL cholesterol [> 35 mg/dl]	58.5	48.2	52.0
LDL cholesterol [≤155 mg/dl]	106.6	110.6	103.2
Total cholesterol [≤200 mg/dl]	180.0	176.4	182.3
Serum creatinine [< 1.2 mg/dl]	0.8	1.0	1.0
CRP [≤0.5 mg/dl]	0.3	1.7	0.4

### The ligands of WNT signaling: WNT3a and WNT5a

**Fig. 2:**
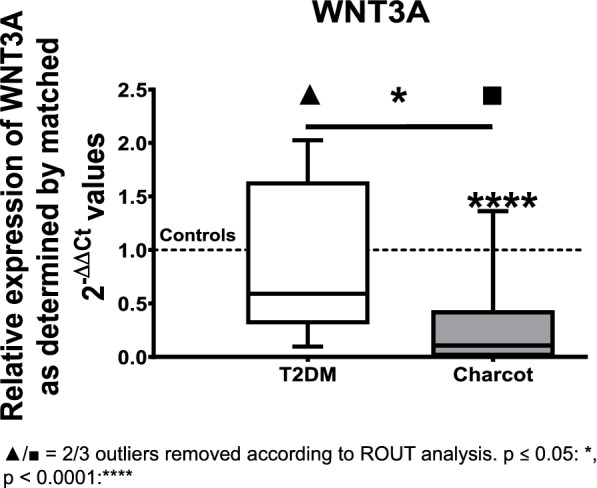
Relative gene expression of WNT3A as determined by matched 2^-ΔΔ*CT*^ values obtained via qPCR in T2DM and Charcot arthropathy patients. The results show a significant decrease of WNT3A expression in Charcot arthropathy patients compared to healthy controls and the type 2 diabetes mellitus group. The data is depicted as boxplot with min./max. Whiskers

Gene expression analysis of WNT3A showed a statistically significant decrease by 89% in the Charcot group compared to the healthy controls (median = 0.1056; *p* < 0.0001), and a notable down-regulation by 82% when compared to T2DM patients not suffering from Charcot arthropathy (median = 0.5916; *p* = 0.0147). The comparison of the T2DM group to the healthy controls did not show significant alterations (see Fig. 2).

**Fig. 3:**
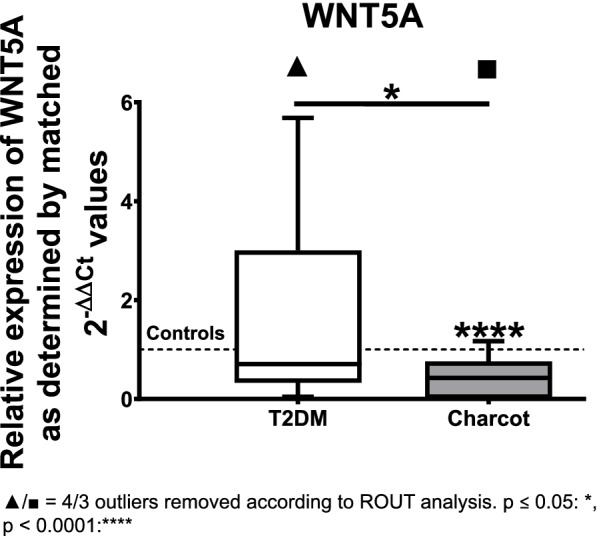
Relative gene expression of WNT5A as determined by matched 2-ΔΔCT values obtained via qPCR in type 2 diabetes mellitus (T2DM) and Charcot arthropathy patients. The results show a significant decrease of WNT5A expression in Charcot arthropathy patients compared to healthy controls and the type 2 diabetes mellitus group. The data is depicted as boxplot with min./max. Whiskers

WNT5A showed a statistically significant decrease by 58% in the Charcot group when compared to the healthy controls (median = 0.4240; *p* < 0.0001), and a notable decrease of gene expression by 40% when compared to T2DM patients (median = 0.7060; *p* = 0.0172). The comparison of T2DM group to the healthy controls did not show significant gene expression changes (see Fig. 3).

### The effectors of Wnt signaling

**Fig. 4:**
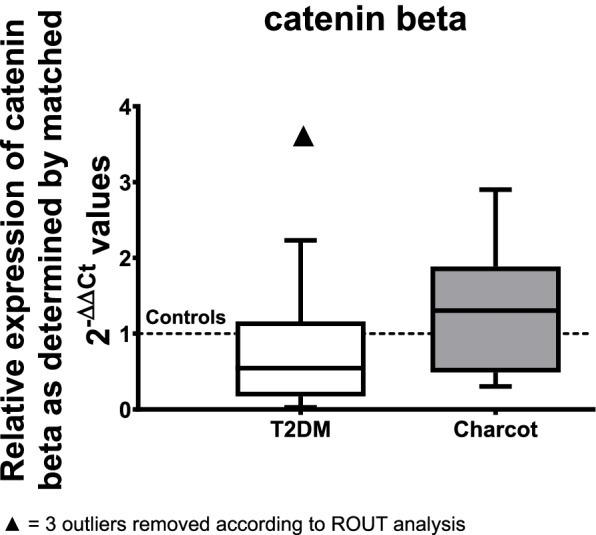
Relative gene expression of catenin beta as determined by matched 2^-ΔΔ*CT*^ values obtained via qPCR in type T2DM and Charcot arthropathy patients. The results show a tendency of reduced expression of catenin beta in the T2DM group. The data is depicted as boxplot with min./max. Whiskers

The gene expression analysis of catenin beta showed a non-significant trend to a decrease by 46% of T2DM patients (median = 0.5435; *p* = 0.3710) when compared to the healthy controls (see Fig. 4). The results regarding Charcot arthropathy did not reveal significant changes compared to the control group.

**Fig. 5:**
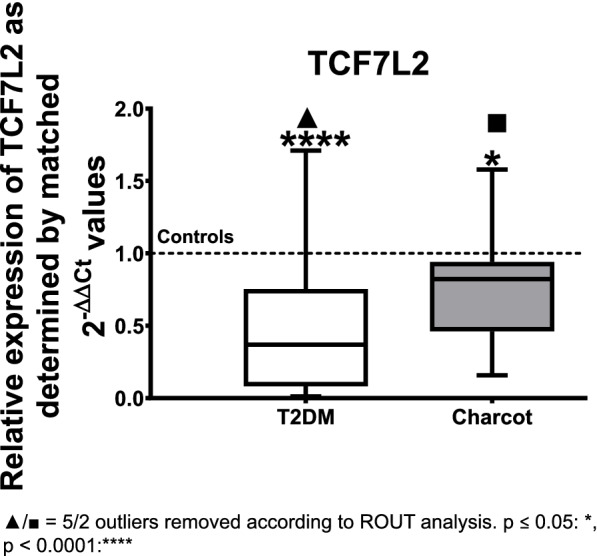
Relative gene expression of TCF7L2 as determined by matched 2^-ΔΔ*CT*^ values obtained via qPCR in T2DM and Charcot arthropathy patients. The results show a distinct down-regulation of 63% in the T2DM patients (*p* < 0.0001). The data is depicted as boxplot with min./max. Whiskers

TCF7L2 revealed a statistically significant decrease by 63% in the T2DM group when compared to the healthy controls (median = 0.3692; *p* < 0.0001), and a statistically significant decrease by 18% when comparing Charcot patients with healthy controls (median = 0.8224; *p* = 0.0136) (see Fig. 5).

### Osteocalcin – a possible target of Wnt-signaling

**Fig. 6:**
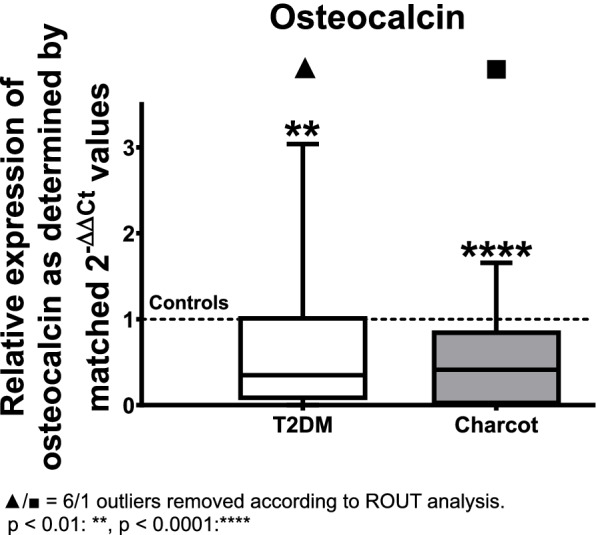
Relative gene expression of osteocalcin as determined by matched 2^-ΔΔ*CT*^ values obtained via qPCR in T2DM and Charcot arthropathy patients. Both T2DM and Charcot arthropathy seem to affect expression of osteocalcin in terms of a significant down-regulation. The data is depicted as boxplot with min./max. Whiskers

The gene expression of osteocalcin was analyzed revealing a statistically significant decrease by 65% in the T2DM group when compared to healthy controls (median = 0.3484; *p* = 0.0019). The expression in the Charcot arthropathy group showed a significant down-regulation by 59% compared to healthy controls (median = 0.4121; *p* < 0.0001) (see Fig. 6).

### Type 1 collagen and fibronectin- components of the extracellular matrix and targets of Wnt signaling

**Fig. 7:**
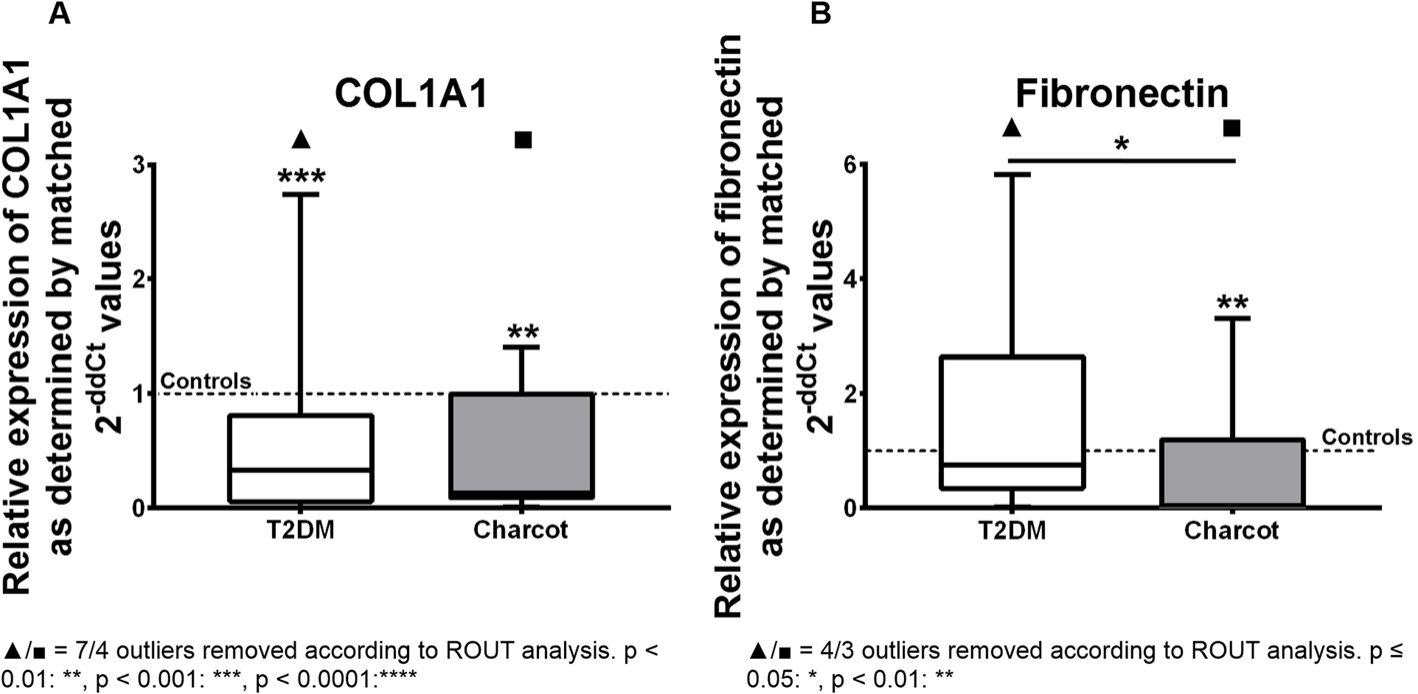
Relative gene expression of type 1 collagen (COL1A1) and fibronectin as determined by matched 2^-ΔΔ*CT*^ values obtained via qPCR in T2DM and Charcot arthropathy patients. Both T2DM and Charcot arthropathy seem to affect expression of these genes in terms of a significant down-regulation. The data is depicted as boxplot with min./max. Whiskers

Furthermore, we analyzed the gene expression of type 1 collagen (COL1A1) and fibronectin. Type 1 collagen showed statistically decrease by 67% in the T2DM group when compared to healthy controls (median = 0.3294; *p* = 0.0006). The expression in the Charcot arthropathy group showed a significant down-regulation by 87% compared to healthy controls (median = 0.1292; *p* = 0.0019). The gene expression of fibronectin was almost abolished in the Charcot group (median = 0.004; *p* = 0.0013) (see Fig. 7).

## Discussion

The Wnt signaling pathway is known to be responsible for the increase of bone density. This is achieved by induction of differentiation and inhibition of apoptosis of osteoblasts [20–24]. Most publications concerning diabetic bone disease describe increased BMD in T2DM, even after adjusting for body mass index and age [2, 3]. However at the same time T2DM is associated with an increased fracture risk [4, 5] and with an impaired bone healing after fracture [7]. Thus dysregulation of Wnt signaling may contribute or be even responsible for these changes.

In this study, we could show a significant downregulation of WNT3A and WNT5A in patients suffering from T2DM induced Charcot arthropathy with the need of surgical treatment. However there was no significant change in the gene expression of both Wnts in T2DM who did not suffer from Charcot arthropathy. The significant finding only in Charcot arthropathy may reflect its more osteodestructive impact on the bone in these patients.

In contrast the analyzed effector of Wnt signaling pathway TCF7L2 and osteocalcin acting downstream showed noticeable similarities between the T2DM and the Charcot arthropathy group. Osteocalcin seems to be involved in regulating insulin production in the endocrine pancreas [29, 30].

The other main effector of the Wnt signaling pathway, catenin beta, did not show significant changes in gene expression in the Charcot group, but showed a tendency to be decreased in T2DM patients. The downregulation of catenin beta in T2DM patients is consistent with the results of previous work [35, 36]. As catenin beta is strongly controlled by post-translational regulation processes, the seeming lack of differential expression patterns may not reflect its true function as an effector of Wnt signaling. Therefore, we analyzed target genes of catenin beta, namely fibronectin and collagen type I alpha 1 chain. Both genes were distinctly down-regulated in both diabetic groups. Fibronectin and type 1 collagen are important components of the organic bone matrix and osteoblasts produce fibronectin during proliferation and differentiation, concomitant with collagen type I production [37–39]. Furthermore fibronectin seems to not only a target but also a modulator of Wnt/ catenin beta signaling [37].

Catenin beta forms a transcription complex with T-cell-specific transcription factors in the nucleus and modulates the transcription of its target genes. One of these co-transcription factors, namely TCF7L2, has been shown to be associated with the development of T2DM. Compared with non-carriers, homozygous carriers of the at-risk alleles show a 2.41-fold risk of developing T2DM ^16^. Mechanisms by which risk alleles of TCF7L2 increase the risk of T2DM include an impaired insulin secretion in response to oral glucose uptake [16, 17]. In isolated adipose tissue of T2DM patients, the expression of TCF7L2 showed a decreased expression level [40]. These findings seem to be congruent with the expression in bone of T2DM patients. In this study, we could show a statistically significant decrease of the transcription of the TCF7L2 gene in the T2DM and also in thoseT2DM patients who suffered from Charcot arthropathy. We observed these changes in TCF7L2 expression in a previous exploratory study in a smaller cohort of diabetic patients (*N* = 10). The gene variants of TCF7L2 are largely found in the intron 3. Therefore, one could hypothesize that these variants impact gene regulation. However, gene regulation is multifactorial and post-translational or other regulatory influences were not assessed.

Interestingly, in osteoblasts, TCF7L2 interacts directly with RUNX family transcription factor 2 (RUNX2), the master gene of osteoblast differentiation. This interaction functionally increases RUNX2 activity [41]. Furthermore, it has been shown that the Wnt signaling activation results in a considerable activation of RUNX2 promoter activity up to 5-fold and 8-fold induction of RUNX2 mRNA in osteoprogenitor cells [42]. In bone, osteocalcin is thought to be involved in mineralization of the bone matrix. Surprisingly, osteocalcin - a protein primarily produced in osteoblasts - also seems to modulate pancreatic beta cell function [26]. In wildtype mice, osteocalcin regulates beta cell gene expression. Isolated islets responded with a 6-fold increase in insulin mRNA expression to the stimulation with exogen osteocalcin [43]. A reduction in osteocalcin serum levels in diabetic patients was described previously and is associated with the risk of increased levels of fasting blood glucose and fat mass [44]. Furthermore, osteocalcin expression seems to be controlled by Wnt signaling [45, 46]. In this study, we could show a statistically significant decrease of osteocalcin gene expression, which may be explained by hampered expression of TCF7L2 and RUNX2, two transcription factors that usually promote osteocalcin synthesis (see Fig. 1).

The molecular etiopathogenesis of Charcot arthropathy is not fully understood. There is a certain number of studies reporting changes in RANKL/RANK/OPG axis (Receptor activator of nuclear factor kappa-Β [ligand]/ osteoprotegerin). The RANKL/RANK/OPG axis orchestrates the interaction of osteoblasts and osteoclasts. Therefore it is of paramount importance for remodeling. In a study by Folestad et al. Charcot arthropathy patients showed initially increased OPG and RANKL levels in peripheral blood samples, which had decreased at the second observation point after 2 years. Interestingly, in this study also soluble members of Wnt signaling were analyzed in blood. The authors describe higher plasma sclerostin and Dkk-1 levels in diabetic control patients. In Charcot arthropathy patients these changes were comparable to the levels of healthy controls. Both molecules have inhibitory effects on Wnt signaling [36]. Other studies do not confirm these findings in peripheral blood [47]. Bruhn-Olszewska et al. showed a correlation between single nucleotide polymorphisms of OPG and Charcot arthropathy [48]. OPG is a target of RUNX2 which in turn seems to be regulated by Wnt signaling [42, 49–51]. Therefore one could hypothesize that changes in gene expression of Wnt signaling may affect the RANKL/RANK/OPG system.

The study has several limitations. First we conducted qPCR to analyze gene expression. Thus statements concerning the corresponding protein-synthesis can only be assumed. Changes on RNA level do not necessarily result in different proteins or even phenotypes. However, several studies showed overall significant correlations between RNA und protein levels [52–55]. The second limitation concerns the size of the Charcot arthropathy group (*N* = 7). As Charcot arthropathy is a spreading, but fortunately overall still rare disease, reported sample sizes in the literature are known to be small. Surgery in Charcot arthropathy is conducted in very few specialized centers only, which moreover narrows the pool of possible sample donors. Nevertheless, our sample size is in line with studies on this special field of research [36, 47, 56].

## Conclusion

For the first time significant changes in gene expression of the Wnt signaling pathway – namely WNT3A, WNT5A, TCF7L2 and osteocalcin- in the bone of patients suffering from T2DM and following Charcot arthropathy could be shown. This may contribute to a better understanding of diabetic bone disease and Charcot arthropathy. With the obtained findings Wnt signaling pathway could serve as a promising novel target in the treatment of T2DM and its feared complication Charcot arthropathy.

## Supplementary Information

**Additional file 1.** Primer Sequences – Supplementary Table 1. WNT3A/5A = Wnt family member 3A/5A, TCF7L2 = transcription factor 7 like 2, COL1A1 = Collagen Type I Alpha 1 Chain.

## Data Availability

The datasets generated and analyzed during the current study are either included in this publication or available from the corresponding author on reasonable request.

## Abbreviations

ANOVA: One-way analysis of variance
BGLAP: Bone gamma-carboxyglutamate; frequently referred to as osteocalcin
BMD: Bone mineral density
BMI: Body mass index
COL1A1: Collagen Type I Alpha 1 Chain
CRP: C-reactive protein
DFG: Deutsche Forschungsgemeinschaft (German Research Foundation)
IGSSE: TUM International Graduate School of Science and Engineering
IRS: Insulin receptor substrate
LRP5/6: Low-density lipoprotein receptor-related protein 5/6
OC: Osteocalcin
OPG: Osteoprotegerin
ROUT: Robust regression and Outlier removal
RANK(L): Receptor activator of nuclear factor kappa-Β (ligand)
RUNX2: Runt-related transcription factor 2
T2DM: type 2 diabetes mellitus
TCF: T cell-specific transcription factor
TCF7L2: Transcription factor 7 like 2
TUM: Technical University of Munich
WNT: Wnt family member
βCat: catenin beta

## References

[1] Prevention CfdCa (2014). National Diabetes Statistics Report: estimates of Diabetes and its burden in the United States.

[2] Schwartz AV, Sellmeyer DE, Ensrud KE, Cauley JA, Tabor HK, Schreiner PJ, Jamal SA, Black DM, Cummings SR (2001). Older women with diabetes have an increased risk of fracture: a prospective study. J Clin Endocrinol Metab.

[3] Vestergaard P (2007). Discrepancies in bone mineral density and fracture risk in patients with type 1 and type 2 diabetes--a meta-analysis. Osteoporosis Int.

[4] Forsen L, Meyer HE, Midthjell K, Edna TH (1999). Diabetes mellitus and the incidence of hip fracture: results from the Nord-Trondelag health survey. Diabetologia.

[5] Schwartz AV (2003). Diabetes mellitus: does it affect bone?. Calcif Tissue Int.

[6] Vilaca T, Schini M, Harnan S, Sutton A, Poku E, Allen IE, et al. The risk of hip and non-vertebral fractures in type 1 and type 2 diabetes: a systematic review and meta-analysis update. Bone. 2020;137:115457. 10.1016/j.bone.2020.115457. Epub 2020 May 29. 10.1016/j.bone.2020.115457 32480023

[7] Retzepi M, Donos N (2010). The effect of diabetes mellitus on osseous healing. Clin Oral Implants Res.

[8] Osima M, Kral R, Borgen TT, Hogestol IK, Joakimsen RM, Eriksen EF, Bjornerem A (2017). Women with type 2 diabetes mellitus have lower cortical porosity of the proximal femoral shaft using low-resolution CT than nondiabetic women, and increasing glucose is associated with reduced cortical porosity. Bone.

[9] Melton LJ, Riggs BL, Leibson CL, Achenbach SJ, Camp JJ, Bouxsein ML, Atkinson EJ, Robb RA, Khosla S (2008). A bone structural basis for fracture risk in diabetes. J Clin Endocrinol Metab.

[10] Chantelau EA, Grutzner G (2014). Is the Eichenholtz classification still valid for the diabetic Charcot foot?. Swiss Med Wkly.

[11] Gouveri E, Papanas N (2011). Charcot osteoarthropathy in diabetes: a brief review with an emphasis on clinical practice. World J Diabetes.

[12] Jeffcoate WJ (2015). Charcot foot syndrome. Diabet Med.

[13] Rogers LC, Frykberg RG, Armstrong DG, Boulton AJ, Edmonds M, Van GH, Hartemann A, Game F, Jeffcoate W, Jirkovska A (2011). The Charcot foot in diabetes. Diabetes Care.

[14] Grant SF, Thorleifsson G, Reynisdottir I, Benediktsson R, Manolescu A, Sainz J, Helgason A, Stefansson H, Emilsson V, Helgadottir A (2006). Variant of transcription factor 7-like 2 (TCF7L2) gene confers risk of type 2 diabetes. Nat Genet.

[15] Ip W, Chiang YT, Jin T (2012). The involvement of the wnt signaling pathway and TCF7L2 in diabetes mellitus: the current understanding, dispute, and perspective. Cell Biosci.

[16] Lyssenko V, Lupi R, Marchetti P, Del Guerra S, Orho-Melander M, Almgren P, Sjogren M, Ling C, Eriksson KF, Lethagen AL (2007). Mechanisms by which common variants in the TCF7L2 gene increase risk of type 2 diabetes. J Clin Invest.

[17] Florez JC, Jablonski KA, Bayley N, Pollin TI, de Bakker PI, Shuldiner AR, Knowler WC, Nathan DM, Altshuler D, Diabetes prevention program research G (2006). TCF7L2 polymorphisms and progression to diabetes in the Diabetes prevention program. N Engl J Med.

[18] Jin T, George Fantus I, Sun J (2008). Wnt and beyond Wnt: multiple mechanisms control the transcriptional property of beta-catenin. Cell Signal.

[19] Geng Y, Ju Y, Ren F, Qiu Y, Tomita Y, Tomoeda M, Kishida M, Wang Y, Jin L, Su F (2014). Insulin receptor substrate 1/2 (IRS1/2) regulates Wnt/beta-catenin signaling through blocking autophagic degradation of dishevelled2. J Biol Chem.

[20] Kulkarni NH, Onyia JE, Zeng Q, Tian X, Liu M, Halladay DL, Frolik CA, Engler T, Wei T, Kriauciunas A (2006). Orally bioavailable GSK-3alpha/beta dual inhibitor increases markers of cellular differentiation in vitro and bone mass in vivo. J Bone Miner Res.

[21] Clement-Lacroix P, Ai M, Morvan F, Roman-Roman S, Vayssiere B, Belleville C, Estrera K, Warman ML, Baron R, Rawadi G (2005). Lrp5-independent activation of Wnt signaling by lithium chloride increases bone formation and bone mass in mice. Proc Natl Acad Sci U S A.

[22] Day TF, Guo X, Garrett-Beal L, Yang Y (2005). Wnt/beta-catenin signaling in mesenchymal progenitors controls osteoblast and chondrocyte differentiation during vertebrate skeletogenesis. Dev Cell.

[23] Bodine PV, Billiard J, Moran RA, Ponce-de-Leon H, McLarney S, Mangine A, Scrimo MJ, Bhat RA, Stauffer B, Green J (2005). The Wnt antagonist secreted frizzled-related protein-1 controls osteoblast and osteocyte apoptosis. J Cell Biochem.

[24] Bodine PV (2008). Wnt signaling control of bone cell apoptosis. Cell Res.

[25] Boyden LM, Mao J, Belsky J, Mitzner L, Farhi A, Mitnick MA, Wu D, Insogna K, Lifton RP (2002). High bone density due to a mutation in LDL-receptor-related protein 5. N Engl J Med.

[26] Zoch ML, Clemens TL, Riddle RC (2016). New insights into the biology of osteocalcin. Bone.

[27] Ferron M, Wei J, Yoshizawa T, Del Fattore A, DePinho RA, Teti A, Ducy P, Karsenty G (2010). Insulin signaling in osteoblasts integrates bone remodeling and energy metabolism. Cell.

[28] Sebastian A, Hum NR, Murugesh DK, Hatsell S, Economides AN, Loots GG (2017). Wnt co-receptors Lrp5 and Lrp6 differentially mediate Wnt3a signaling in osteoblasts. PLoS One.

[29] Okamoto M, Udagawa N, Uehara S, Maeda K, Yamashita T, Nakamichi Y, Kato H, Saito N, Minami Y, Takahashi N (2014). Noncanonical Wnt5a enhances Wnt/beta-catenin signaling during osteoblastogenesis. Sci Rep.

[30] Almeida M, Han L, Bellido T, Manolagas SC, Kousteni S (2005). Wnt proteins prevent apoptosis of both uncommitted osteoblast progenitors and differentiated osteoblasts by beta-catenin-dependent and -independent signaling cascades involving Src/ERK and phosphatidylinositol 3-kinase/AKT. J Biol Chem.

[31] Kerner W, Bruckel J, German Diabetes A (2014). Definition, classification and diagnosis of diabetes mellitus. Exp Clin Endocrinol Diabetes.

[32] Rosenbaum AJ, DiPreta JA (2015). Classifications in brief: Eichenholtz classification of Charcot arthropathy. Clin Orthop Relat Res.

[33] Livak KJ, Schmittgen TD (2001). Analysis of relative gene expression data using real-time quantitative PCR and the 2(−Delta Delta C(T)) method. Methods.

[34] Haug AT, Braun KF, Ehnert S, Mayer L, Stockle U, Nussler AK, Pscherer S, Freude T (2014). Gene expression changes in cancellous bone of type 2 diabetics: a biomolecular basis for diabetic bone disease. Langenbeck's Arch Surg.

[35] Gaudio A, Privitera F, Pulvirenti I, Canzonieri E, Rapisarda R, Fiore CE (2014). The relationship between inhibitors of the Wnt signalling pathway (sclerostin and Dickkopf-1) and carotid intima-media thickness in postmenopausal women with type 2 diabetes mellitus. Diab Vasc Dis Res.

[36] Folestad A, Alund M, Asteberg S, Fowelin J, Aurell Y, Gothlin J, Cassuto J (2015). Role of Wnt/beta-catenin and RANKL/OPG in bone healing of diabetic Charcot arthropathy patients. Acta Orthop.

[37] Yang C, Wang C, Zhou J, Liang Q, He F, Li F, Li Y, Chen J, Zhang F, Han C (2020). Fibronectin 1 activates WNT/beta-catenin signaling to induce osteogenic differentiation via integrin beta1 interaction. Lab Investig.

[38] Gradl D, Kuhl M, Wedlich D (1999). The Wnt/Wg signal transducer beta-catenin controls fibronectin expression. Mol Cell Biol.

[39] Cho SW, Yang JY, Sun HJ, Jung JY, Her SJ, Cho HY, Choi HJ, Kim SW, Kim SY, Shin CS (2009). Wnt inhibitory factor (WIF)-1 inhibits osteoblastic differentiation in mouse embryonic mesenchymal cells. Bone.

[40] Cauchi S, Meyre D, Dina C, Choquet H, Samson C, Gallina S, Balkau B, Charpentier G, Pattou F, Stetsyuk V (2006). Transcription factor TCF7L2 genetic study in the French population: expression in human beta-cells and adipose tissue and strong association with type 2 diabetes. Diabetes.

[41] McCarthy TL, Centrella M (2010). Novel links among Wnt and TGF-beta signaling and Runx2. Mol Endocrinol.

[42] Gaur T, Lengner CJ, Hovhannisyan H, Bhat RA, Bodine PV, Komm BS, Javed A, van Wijnen AJ, Stein JL, Stein GS (2005). Canonical WNT signaling promotes osteogenesis by directly stimulating Runx2 gene expression. J Biol Chem.

[43] Ferron M, Hinoi E, Karsenty G, Ducy P (2008). Osteocalcin differentially regulates beta cell and adipocyte gene expression and affects the development of metabolic diseases in wild-type mice. Proc Natl Acad Sci U S A.

[44] Kindblom JM, Ohlsson C, Ljunggren O, Karlsson MK, Tivesten A, Smith U, Mellstrom D (2009). Plasma osteocalcin is inversely related to fat mass and plasma glucose in elderly Swedish men. J Bone Miner Res.

[45] Neve A, Corrado A, Cantatore FP (2013). Osteocalcin: skeletal and extra-skeletal effects. J Cell Physiol.

[46] Lee NK, Sowa H, Hinoi E, Ferron M, Ahn JD, Confavreux C, Dacquin R, Mee PJ, McKee MD, Jung DY (2007). Endocrine regulation of energy metabolism by the skeleton. Cell.

[47] Connors JC, Hardy MA, Kishman LL, Botek GG, Verdin CJ, Rao NM, Kingsley JD (2018). Charcot pathogenesis: a study of in vivo gene expression. J Foot Ankle Surg.

[48] Bruhn-Olszewska B, Korzon-Burakowska A, Wegrzyn G, Jakobkiewicz-Banecka J (2017). Prevalence of polymorphisms in OPG, RANKL and RANK as potential markers for Charcot arthropathy development. Sci Rep.

[49] Boyce BF, Xing L, Chen D (2005). Osteoprotegerin, the bone protector, is a surprising target for beta-catenin signaling. Cell Metab.

[50] Kieslinger M, Folberth S, Dobreva G, Dorn T, Croci L, Erben R, Consalez GG, Grosschedl R (2005). EBF2 regulates osteoblast-dependent differentiation of osteoclasts. Dev Cell.

[51] Mikasa M, Rokutanda S, Komori H, Ito K, Tsang YS, Date Y, Yoshida CA, Komori T (2011). Regulation of Tcf7 by Runx2 in chondrocyte maturation and proliferation. J Bone Miner Metab.

[52] Gry M, Rimini R, Stromberg S, Asplund A, Ponten F, Uhlen M, Nilsson P (2009). Correlations between RNA and protein expression profiles in 23 human cell lines. BMC Genomics.

[53] Fu N, Drinnenberg I, Kelso J, Wu JR, Paabo S, Zeng R, Khaitovich P (2007). Comparison of protein and mRNA expression evolution in humans and chimpanzees. PLoS One.

[54] Shankavaram UT, Reinhold WC, Nishizuka S, Major S, Morita D, Chary KK, Reimers MA, Scherf U, Kahn A, Dolginow D (2007). Transcript and protein expression profiles of the NCI-60 cancer cell panel: an integromic microarray study. Mol Cancer Ther.

[55] Greenbaum D, Colangelo C, Williams K, Gerstein M (2003). Comparing protein abundance and mRNA expression levels on a genomic scale. Genome Biol.

[56] Pasquier J, Thomas B, Hoarau-Vechot J, Odeh T, Robay A, Chidiac O, Dargham SR, Turjoman R, Halama A, Fakhro K (2017). Circulating microparticles in acute diabetic Charcot foot exhibit a high content of inflammatory cytokines, and support monocyte-to-osteoclast cell induction. Sci Rep.

