# Supplementary material for: Dysregulation of Wnt signaling in bone of type 2 diabetes mellitus and diabetic Charcot arthropathy
Source: BMC Musculoskelet Disord. 2022 Apr 18;23:365. doi: 10.1186/s12891-022-05314-9 (PMC9017014; doi:10.1186/s12891-022-05314-9)
Supplement: Supplementary file 1 — Additional file 1. Primer Sequences – Supplementary Table 1. WNT3A/5A = Wnt family member 3A/5A, TCF7L2 = transcription factor 7 like 2, COL1A1 = Collagen Type I Alpha 1 Chain. [file 12891_2022_5314_MOESM1_ESM.docx]

| Primer | Forward sequence | Reverese sequence |
| --- | --- | --- |
| WNT3A | 5‘–AAC TGC ACC ACC GTC CAC–3‘ | 5‘–AAG GCC GAC TCC CTG GTA–3‘ |
| WNT5A | 5‘–ATT GTA CTG CAG GTG TAC CTT AAA AC–3‘ | 5‘–CCC CCT TAT AAA TGC AAC TGT TC–3‘ |
| Catenin BETa | 5‘–GCT TTC AGT TGA GCT GAC CA–3‘ | 5‘–CAA GTC CAA GAT CAG CAG TCT C–3‘ |
| TCF7L2 | 5‘–TTG ACC GAC AGA CTT TAT GGT G–3‘ | 5‘–TGT ATG TAG CGA ACG CAC TTT T–3‘ |
| Osteocalcin | 5‘–TGA GAG CCC TCA CAC TCC TC–3‘ | 5‘–ACC TTT GCT GGA CTC TGC AC–3‘ |
| Fibronectin | 5‘–CCC CAT TCC AGG ACA CTT CTG–3‘ | 5‘–GCC CAC GGT AAC AAC CTC TT–3‘ |
| COL1A1 | 5‘–GGG ATT CCC TGG ACC TAA AG–3‘ | 5‘GGA ACA CCT CGC TCT CCA–3‘ |
| Actin Beta | 5‘–ACA GAG CCT CGC CTT TGC CGA T–3‘ | 5‘–GCG AAG CCG GCC TTG CAC AT–3‘ |

**Primer Sequences – Supplementary Table 1.** WNT3A/5A = Wnt family member 3A/5A, TCF7L2 = transcription factor 7 like 2, COL1A1 = Collagen Type I Alpha 1 Chain.
